# CX-01 mitigates trauma-induced acute kidney injury and improves survival in a combat-relevant polytrauma rat model

**DOI:** 10.3389/fphar.2026.1834668

**Published:** 2026-07-02

**Authors:** Zhangsheng Yang, Caroline Gusson Shimoura, Heaven D. Sessions, Dustin M. Kneifel, Jeanette Rocha, Kassandra Gonzalez, Robert P. Willis, Emily M. Corbin, Venkata Yellepeddi, Brian J. Kirkwood, Jose Salinas, Andrew D. Meyer

**Affiliations:** 1 Expeditionary Medical Systems Department, United States Army Institute of Surgical Research, San Antonio, TX, United States; 2 Research Support Directorate, United States Army Institute of Surgical Research, San Antonio, TX, United States; 3 Division of Clinical Pharmacology, Department of Pediatrics, Spencer Fox Eccles School of Medicine, University of Utah, Salt Lake City, UT, United States; 4 Long School of Medicine, the University of Texas Health Science Center, San Antonio, TX, United States

**Keywords:** acute kidney injury, coagulation profile, CX-01, high mobility group box 1, inflammation, polytrauma, rat, survival

## Abstract

Previous studies have shown that polytrauma may initiate a systemic inflammatory response that drives acute kidney injury (AKI), multiple organ failure (MOF), and high mortality. However, beyond supportive care, pharmacological treatments for trauma-induced AKI are lacking, which represents a serious unmet clinical need. Previous work from our group identified the inflammatory mediator high mobility group box 1 (HMGB1) as a promising therapeutic target to mitigate MOF and AKI. We have shown that HMGB1 levels correlate with organ failure, such as AKI, acute respiratory distress syndrome (ARDS), and MOF. In this study, we investigated CX-01, a modified heparin and HMGB1 inhibitor, to determine if it can reduce AKI and improve survival in a rat polytrauma model. Male Sprague-Dawley (SD) rats underwent polytrauma consisting of soft tissue injury, fibula fracture and pressure-controlled hemorrhage (MAP 55 mmHg) for 2 hours followed by whole blood resuscitation to maintain MAP at 90 mmHg for 30 min. Animals were randomly assigned to either a vehicle control group (vehicle, n = 9), or a therapeutic treatment group which received CX-01 (25 mg/kg, n = 8) at specified intervals over a 72-h observation period. Vital signs, hemodynamics, blood chemistry, inflammation, and tissue damage were collected and analyzed. Treatment with CX-01 significantly increased survival over the 72-h study period (87.5% vs. 33.3%, *p* < 0.05). The CX-01 treatment group showed reduced trauma-induced AKI, as evidenced by significantly improved glomerular filtration rate (GFR) (0.87 vs. 0.56, *p* < 0.05) at 2.5 h post-injury compared to controls. Histological evaluations indicated that CX-01 treatment markedly reduced total kidney injury scores from 2.86 (0.29–4.14) to 0.86 (0.29–1.29). At 2.5 h post-injury, the treatment significantly attenuated trauma-induced lactate elevation. CX-01 therapy significantly decreased the release of HMGB1 and several inflammatory cytokines including MCP-1, RANTES, and GRO/KC (*p* < 0.05 for all) at 2.5 h post-injury. Furthermore, CX-01 treatment did not alter the coagulation profile in these polytrauma animals when compared to vehicle control. These results demonstrate that CX-01 treatment might reduce trauma-induced AKI and improve survival in a clinically relevant rat model. Future studies should focus on validation of this approach in large animal models or advancing CX-01 toward clinical trials in trauma patients.

## 1 Introduction

Severe trauma can result in serious organ dysfunction such as acute kidney injury (AKI) which can lead to multiple organ failure (MOF) and death if left untreated. In civilian intensive care unit (ICU) studies AKI has been shown to occur in trauma patients with incidence rates ranging from 6% to 56% depending on the specific populations and admission criteria (Bagshaw et al., 2008; Eriksson et al., 2015; Haines et al., 2018; Podoll et al., 2013; Bihorac et al., 2010; Schiefer et al., 2023). In the military setting, AKI is also frequently encountered among combat casualties. Stewart and colleagues reported that 12.5% of service members in Iraq or Afghanistan (474 out of 3,807) suffered from AKI (Stewart et al., 2016). Furthermore, the incidence of AKI tends to rise with an increasing injury severity score (ISS). Elterman et al. reported that for the warfighters with a median ISS of 22, the AKI incidence can be up to 21% (Elterman et al., 2015). The onset of AKI commonly occurs early during hospital admission, with up to 34.3% of patients developing the condition within the first 2 days of hospitalization (Heegard et al., 2015).

Numerous risk factors from traumatic injury contribute to the development of AKI, including systemic inflammation, hemorrhagic shock, massive transfusion, rhabdomyolysis, abdominal compartment syndrome and major surgery (Eriksson et al., 2015; Bihorac et al., 2013; Lord et al., 2014). Trauma-induced AKI is caused by inadequate renal perfusion, direct renal injury (Harrois et al., 2017; Harrois et al., 2018; Zimmerman and Shen, 2013), as well as the patient’s inflammatory cytokine response (Bihorac et al., 2013). These circulating inflammatory cytokines, in conjunction with chemokines, activate leukocytes and adhesion molecules, leading to distant organ immune cell infiltration and subsequent dysfunction in the kidney (Okusa, 2010; Awad et al., 2009).

After severe trauma, the inflammatory response to blood loss and tissue damage can start immediately, leading to instantaneous release of damage-associated molecular patterns (DAMPs), such as high mobility group box 1 (HMGB1) (also known as amphoterin) protein, histones, heat-shock proteins, and other inflammatory mediators (Levy et al., 2007; Laird et al., 2014; Relja et al., 2018). The activation of DAMPs further triggers a secondary inflammatory cascade involving cytokines, complement, reactive oxygen species (ROS), and endothelial cell damage, that can contribute to coagulopathy, endotheliopathy, complementopathy, and ultimately organ failure (Yang et al., 2023a; Yang et al., 2022a; Kozlov and Lancaster, 2017; Moore et al., 2021). Indeed, previous studies from our group and others demonstrate that HMGB1, a key DAMP, is rapidly elevated in circulation after traumatic injury in both combat casualties and preclinical animal models (Yang et al., 2022b; Peltz et al., 2009; Cohen et al., 2009). Moreover, an increase in HMGB1 activation correlates with trauma-induced organ failure such as AKI, acute respiratory distress syndrome (ARDS), and MOF in a swine model of smoke inhalation and burn injury (Young et al., 2023; Yang et al., 2024).

AKI is an independent risk predictor of mortality in civilian trauma patients and military casualties (Bagshaw et al., 2008; Haines et al., 2018; Stewart et al., 2016). While numerous studies have aimed to prevent or reduce AKI, there is still no FDA-approved pharmacological therapy to treat this devasting disease. The management of AKI remains largely dependent on supportive strategies and renal replacement therapy (Group, 2012). Recently, accumulating evidence suggests that inflammation might be contributing to trauma-induced AKI, therefore therapeutics that target inflammation might mitigate trauma-induced AKI (Yang et al., 2024; Zhou, 2023; Messerer et al., 2021; Panizo et al., 2015). Given this, the aim of this study was to test whether a therapeutic treatment with a low-anticoagulant heparin derivative (2-O, 3-O desulfated heparin) known as CX-01 can reduce trauma-induced AKI and increase survival in a rat polytrauma model.

## 2 Materials and methods

### 2.1 Animal study

Research was conducted in compliance with Animal Welfare Act, the implementing Animal Welfare regulations, and the principles of the Guide for the Care and Use of Laboratory Animals. The Institutional Animal Care and Use Committee approved all research conducted in this study. The facility where this research was conducted is fully accredited by the AAALAC International.

### 2.2 Surgical procedures and injury model in rats

This study used specific-pathogen-free adult male Sprague-Dawley rats (10–15 weeks old), weighing 300–500 g (Charles River Laboratories, Wilmington, MA). All the animals were given proper analgesics during the entire study, as buprenorphine SR-Lab (1.2 mg/kg) was given prior to the surgical cannulations, and additional buprenorphine HCl (0.01–0.05 mg/kg) was administered as needed at later time points. Under anesthesia, the carotid artery, femoral artery and femoral vein were cannulated in all rats. After stabilization, polytrauma injuries were performed consisting of soft tissue injury, fibula fracture, and pressure-controlled hemorrhage as described previously (Xiang et al., 2022). The soft tissue injury was induced via clamping the retrofemoral tissue group for 30 s using an angled Kelly clamp at the first notch, and fibula facture was conducted using a 15-gauge needle penetrated through the skin and fascia between the tibia and fibula. The fibula fracture was intentionally left untreated (un-splinted) to mimic a severe battlefield or civilian crush injury where immediate orthopedic fixation is unavailable, thereby ensuring a sustained release of DAMPs to drive the systemic inflammatory response. After the extremity trauma, hemorrhage was conducted by withdrawing blood from the femoral artery at a rate of 0.5 mL/min using an automatic infusion/withdrawal syringe pump (Harvard Apparatus, model PHD 22/2000) until the mean arterial pressure (MAP) decreased to 55 mmHg. The MAP was maintained at 55 mmHg (±3 mmHg) for a 2-h period to induce hemorrhagic shock by either withdrawing additional blood or infusing back the shed blood (Xiang et al., 2022). After 2 hours of hemorrhagic shock, all the animals were subjected to a donor whole blood resuscitation to return the MAP to 90–100 mmHg [rat normal MAP is 90–120 mmHg (Banek et al., 2021; Ramirez et al., 2023)] for 30 min. The animals were then returned to their housing for 72 h post-injury monitoring or until death or euthanasia. For humane euthanasia, animals underwent exsanguination under deep anesthesia via the carotid artery or abdominal aorta, or received an intravenous (I.V.) or intracardiac (I.C.) injection of Fatal-Plus® euthanasia solution (at least 150 mg/kg body weight).

### 2.3 Therapeutic treatments and data collection

**FIGURE 1:**
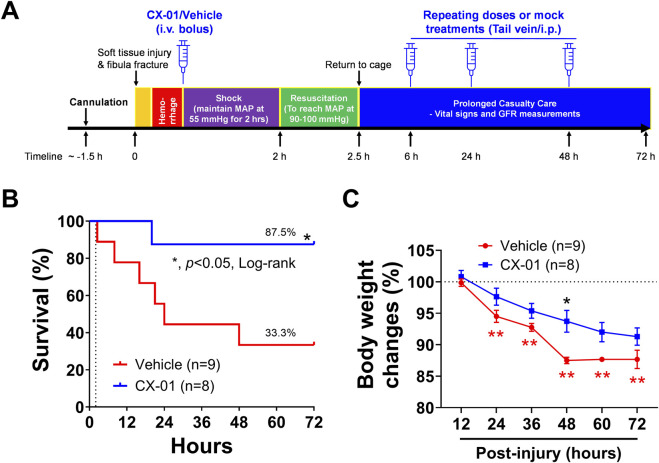
CX-01 treatment increases survival in a rat model of polytrauma. **(A)** The experimental design and timeline of CX-01 therapeutic treatment on rat polytrauma model. The Sprague-Dawley (SD) rats were subjected to a polytrauma consisting of soft tissue injury, fibula fracture, and pressure-controlled hemorrhage, and followed by resuscitation with donor blood to maintain a mean arterial pressure (MAP) of 90–100 mmHg for 30 min. After resuscitation, the rats were allowed to recover and returned to their cages for observation. The animals were randomly assigned to one of two groups: a vehicle control group that received normal saline (n = 9) or a CX-01 treatment group (n = 8). The first dose of CX-01 (25 mg/kg) was administered as an intravenous (i.v.) bolus immediately after hemorrhage. Three subsequent doses (25 mg/kg each) were administered via tail vein or intraperitoneal (i.p.) injection. All animals were monitored for 72 h post-injury or until death or the need for early euthanasia. **(B)** CX-01 treatment significantly increased survival compared to the vehicle control group. The data were analyzed using a Log-rank test. *, *p* < 0.05. **(C)** The body weight changes in the vehicle control and CX-01 treated animals were monitored two times per day after injury. Statistical analyses were performed by the linear mixed-effect model for repeated measures. *, *p* < 0.05 (black), the value of individual time point of vehicle control vs. CX-01. An unpaired Mann-Whitney rank test was used to compare individual time points to baseline within the injury group (**, *p* < 0.01, red).

The animals were randomly assigned to either vehicle control (vehicle, n = 9), or therapeutic treatment with an immunotherapeutic agent (CX-01, 25 mg/kg, n = 8). Four doses of CX-01 or mock treatments were administered in total. The first dose (25 mg/kg) of CX-01 was given intravenously immediately after hemorrhage, and three repeating doses were administrated at 6-, 24-, and 48- hours after extremity trauma via tail vein injection or intraperitoneal (i.p.) injection (Figure 1). The vital signs and hemodynamics were recorded by a PowerLab system (ADInstruments, Colorado spring, CO) and a PhysioSuite system (Kent Scientific, Torrington, CT). The blood samples were collected at baseline (BL), 2-, end of resuscitation (EOR, 2.5-), 6-, 24-, 48-, and 72 h post-injury (Figure 1). Blood gases and chemistry were analyzed using an i-STAT (Abbott Laboratories, Abbott Park, Illinois, USA). The tissue samples were collected at necropsy at the end of study.

### 2.4 Glomerular filtration rate measurement

The glomerular filtration rate (GFR) was measured in conscious rats using a transdermal MX GFR monitor (MediBeacon) as described previously (Xiang et al., 2022; Xiang et al., 2023). The rats were anesthetized by isoflurane, and a double-sided adhesive patch was attached to the skin, and a small device with two light-emitting diodes was attached to this adhesive patch. One diode’s purpose is to excite FITC-sinistrin at 480 nm while another diode detects the light signal at 521 nm in a transcutaneous manner. Rats were placed in a mesh wrap (to ensure that the device remains affixed to the back) and allowed to wake up (approximately 2 min) and ambulate. Prior to waking up, a bolus of FITC-sinistrin was injected into the vein/artery catheter at 30–50 mg/kg body weight for rats. Two to three hours later, the device along with the jacket and adhesive patch was removed from the rat. The device was connected to a computer for data storage and analysis of excretion kinetics of FITC-sinistrin (Bihorac et al., 2013). GFR was calculated based on the formula: GFR (ml/min per 100 g Bw) = 31.26 (ml/100 g Bw)/t½. t½ is the half-life of FITC-sinistrin.

### 2.5 ELISA assay and bioplex assay

The quantitative levels of inflammatory mediators HMGB1 (CAT#30164033, Tecan, Switzerland) and myeloperoxidase (MPO) (CAT#HK105, Hycult Biotech, Netherlands) in the plasma samples were measured by using commercial ELISA kits according to the manufacturer’s instructions. The inflammatory cytokines RANTES (regulated on activation, normal T cell expressed and secreted, also known as CCL-5), GRO/KC (growth-regulated oncogene/keratinocyte chemoattractant), MCP-1 (monocyte chemoattractant protein-1), MIP-1α (macrophage inflammatory protein-1 alpha, also known as CCL-3), M-CSF (macrophage colony-stimulating factor), IL-4, IL-5 and IL-10 in the plasma samples were measured by using an customized bio-plex kit (CAT#17011106, Bio-Rad, Hercules, CA) or Bio-Plex Pro™ Rat Cytokine 23-Plex (CAT#12005641, Bio-Rad, Hercules, CA), and performed following the manufactures’ recommendations.

To quantify kidney injury molecule-1 (KIM-1) in kidney tissue homogenates, approximately 100 mg of frozen kidney tissue was harvested. The tissue was lysed on ice in 400–600 µL of M-PER™ Mammalian Protein Extraction Reagent (CAT#78503, Thermo Scientific, Waltham, MA, USA) supplemented with a protease inhibitor cocktail (CAT#P8340, MilliporeSigma, Burlington, MA, USA). The tissue was then homogenized for 30–60 s using a rotor-stator homogenizer, followed by sonication for three cycles of 10 s on and 10 s off. After centrifugation at 14,000 × g, the resulting supernatant was harvested and aliquoted. A bicinchoninic acid (BCA) protein assay was used to measure the total protein concentration. The levels of KIM-1 in the tissue were measured using a commercially available ELISA kit (Cat#RKM100, R&D Systems, Minneapolis, MN, USA) according to the manufacturer’s instructions. The final values were normalized to the total protein concentration of each sample.

### 2.6 Histopathological evaluation

The kidney tissues were collected at necropsy and fixed in 10% neutral buffered formalin for a minimum of 48 h. After fixation, transverse sections were routinely processed and embedded in paraffin. The formalin-fixed, paraffin embedded tissue sections were cut via microtome by a certified histotechnologist at 5 µm thickness, mounted onto glass slides, and routinely stained with hematoxylin and eosin (H&E) for histopathologic evaluation. Photomicrographs of entire tissue sections from each rat were captured with a 10× or 40× objective using an Olympus microscope (Olympus, BX53, Tokyo, Japan). Representative images from each group are presented (Figure 3B). The histologic slides were evaluated by a board-certified veterinary pathologist who was blinded to the treatment group. Each entire kidney was evaluated using an Olympus light microscope (Olympus, BX41, Tokyo, Japan) for (Bagshaw et al., 2008) severity: 0 = normal histology; 1 = minimal alteration; 2 = mild; 3 = moderate; 4 = severe, and (Eriksson et al., 2015) extent of injury: 0: absent; 1: <25%; 2: 25%–50%; 3: 50%–75%; 4: >75%. Parameters evaluated included two types of necrosis (infarct and acute tubular necrosis), three indicators of degeneration (tubular epithelial degeneration, proteinosis, and granular and/or tubular casts), vasa recta congestion, interstitial hemorrhage, glomerular capillary thrombosis, interstitial lymphoplasmacytic infiltrates, and other tubular findings (intracytoplasmic hyaline droplets, mineralization of the proximal convoluted tubule). Other individual findings were recorded but not scored, including bacterial infection within infarcted tissue with surrounding neutrophilic inflammation (n = 1), focal marked neutrophilic glomerulitis spatially associated with glomerular capillary thrombosis (n = 1), rare interstitial extramedullary hematopoiesis (n = 2), and glomerular basement membrane thickening (n = 1).

A semi-quantitative score was utilized to assess kidney injury, following previously described methods (Wang X. et al., 2015; Murphy et al., 2009; Simovic et al., 2023). This score was determined by summing the severity and extent of injury for each subject in both the vehicle control and CX-01 treated groups, specifically accounting for renal degeneration/necrosis, and congestion/hemorrhage. Additionally, a specific semi-quantitative analysis was performed to calculate a kidney proteinosis score, similarly derived by summing the severity and extent of the observed proteinaceous casts.

### 2.7 Coagulation profile by STAGO

Platelet-free plasma was prepared by sequential centrifugation at 2,500 *g* for 15 min at room temperature twice and then aliquoted into 1.5-mL Nunc® Cryotubes® (Sigma) for storage at −80 °C. Using manufacturer’s directions, the Diagnostica STAGO Compact Max® was used to measure prothrombin time (PT), activated partial thromboplastin time (aPTT), fibrinogen, and D-dimer. For all assays, samples were treated prior to analysis with 50 μL of Dade® hepzyme per 500 μL sample to reverse residual heparin in the sample after being incubated at 37 °C for one to 2 minutes.

### 2.8 Statistical analysis

Chemistry, vital signs, and blood gas data in Table 1 were presented as median and interquartile range (25%–75% percentiles), and a Mann–Whitney U test was applied for the statistical analyses. Longitudinal data were presented as mean and standard error of the mean (SEM). A mixed-effects model was used for longitudinal data; however, it should be noted that this model assumes missing data is missing at random (MAR). Because missingness in this study was driven by mortality (informative missingness), data at later time points are subject to survivor bias. An unpaired Mann-Whitney rank test was used to compare individual time points to baseline within the injury group. The histological scoring was analyzed by unpaired Mann-Whitney rank test, and the data are presented as median and interquartile range. Statistical significance was determined with a two-sided *p* < 0.05. Statistical analyses were performed using GraphPad Prism 10.4.0 (GraphPad Software, San Diego, CA).

**TABLE 1:** The impact of chemistry, blood gas, and vital sign of the animals with CX-01 treatment.

Parameters	Groups	BL	EOS	EOR	24 h	48 h	72 h	References ranges (Banek et al., 2021; Ramirez et al., 2023; He et al., 2017; Subramanian et al., 2013)
MAP (mmHg)	Vehicle	90.00 (87.30–96.14)	55.10 (55.00–56.50)***	95.77 (92.00–99.50)	79.10 (60.80–86.00)**	73.00 (66.00–95.90)	66.00 (65.00–91.00)	90–120
	CX-01	93.00 (91.15–97.15)	56.70 (55.55–56.98)	98.00 (91.75–105.00)	89.30 (80.65–94.95)	88.80 (84.76–100.88)	87.20 (85.00–87.40)	
**HR (bpm)**	Vehicle	398.00 (373.50–407.50)	390 (365.50–423.50)	402.00 (380.25–420.50)	410.50 (361.25–420.00)	399.00 (381.00–403.00)	416.00 (375.00–432.00)	300–450
	CX-01	346.50 (331.80–392.25)	348.50 (337.25–388.00)	380.55 (370.08–406.75)	383.00 (297.00–410.70)	351.50 (265.48–400.75)	368.00 (315.00–408.00)	
SPO2 (%)	Vehicle	94.00 (90.50–99.00)	86.00 (80.50–94.50)	88.00 (86.00–95.00)	98.00 (89.75–98.75)	97.50 (89.50–98.75)	97.00 (87.00–99.00)	>95%
	CX-01	97.50 (94.00–99.00)	87.00 (84.00–96.00)	96.00 (90.00–99.00)	98.00 (97.00–99.00)	97.00 (94.00–99.00)	97.00 (94.00–99.00)	
PaO2 (mmHg)	Vehicle	61.00 (58.00–72.00)	74.00 (68.00–83.00)	75.00 (65.00–84.00)	77.00 (37.50–97.75)	81.00 (74.00–91.00)*	68.00 (51.00–79.00)	80–105
	CX-01	57.50 (43.00–72.00)	72.00 (60.25–80.75)	67.50 (46.75–87.50)	78.00 (73.00–81.00)	83.00 (78.00–83.00)	60.50 (48.00–73.00)	
PCO2 (mmHg)	Vehicle	44.30 (30.35–51.15)	36.70 (34.40–44.95)	41.50 (35.65–47.80)	41.50 (33.30–46.90)	33.70 (23.13–39.33)	40.20 (33.50–43.60)	35–45
	CX-01	53.20 (47.20–59.55)	43.80 (36.75–60.10)	49.10 (41.65–54.55)	46.55 (34.80–58.30)	38.30 (35.30–40.70)	48.30 (39.73–76.30)	
HCO3 (mmol/L)	Vehicle	**26.40 (22.70–27.70)**	20.80 (18.45–21.65)*	**25.95 (23.05–27.68)**	26.20 (24.20–34.50)	23.25 (17.45–28.30)	25.60 (23.50–29.10)	22–26
	CX-01	**29.05 (28.15–29.60)††**	**23.25 (21.90–24.55)††**	**28.80 (26.80–29.35)†**	33.00 (31.00–33.60)	29.05 (28.55–33.73)	28.80 (27.10–29.50)	
TCO2 (mmol/L)	Vehicle	**26.00 (22.50–27.00)**	21.00 (19.50–21.00)*	**24.00 (22.50–26.50)**	25.00 (23.50–30.25)	22.00 (23.00–27.00)	25.00 (23.00–27.00)	24–29
	CX-01	**28.00 (27.25–28.75)††**	**23.00 (21.00–24.00)†**	**27.00 (25.25–28.00)†**	29.00 (28.75–30.00)	27.50 (25.75–30.25)	27.00 (26.00–28.00)	
pH	Vehicle	7.36 (7.32–7.38)	7.33 (7.25–7.39)	7.39 (7.38–7.43)*	7.47 (7.47–7.48)	7.47 (7.44–7.49)	7.43 (7.41–7.45)	7.35–7.45
	CX-01	7.34 (7.28–7.38)	7.30 (7.17–7.38)	**7.35 (7.33–7.41)†**	7.44 (7.41–7.50)	7.47 (7.43–7.53)	7.46 (7.38–7.50)	
Na^+^ (mmol/L)	Vehicle	139.00 (137.00–146.50)	141.00 (140.00–146.00)	140.00 (138.00–143.00)	139.00 (136.50–140.50)	141.50 (137.25–142.75)	139.00 (137.00–142.00)	138–146
	CX-01	138.50 (137.00–140.00)	141.0 (140.00–144.25)	140.00 (138.00–141.75)	140.00 (136.50–142.50)	140.00 (139.25–141.25)	139.00 (138.00–141.00)	
K^+^(mmol/L)	Vehicle	3.90 (3.15–4.30)	4.70 (4.00–5.45)*	4.70 (4.10–5.30)**	4.00 (3.05–7.50)	4.15 (3.83–6.73)	3.60 (3.50–4.70)	3.5–4.9
	CX-01	4.50 (4.30–4.60)	4.85 (3.90–5.00)	4.85 (4.55–5.08)	4.20 (3.60–5.15)	4.15 (3.70–4.58)	4.20 (3.90–4.60)	
Chloride (mmol/L)	Vehicle	105.00 (102.50–109.50)	104.00 (102.00–108.50)	105.00 (103.00–108.00)	105.00 (100.00–107.50)	108.50 (105.75–109.00)	105.00 (102.00–106.00)	98–109
	CX-01	104.00 (103.00–105.00)	108.50 (101.50–109.75)	105.00 (104.25–106.75)	101.50 (78.15–107.00)	100.00 (98.00–105.25)	101.00 (101.00–102.00)	
Glucose (mmol/L)	Vehicle	171.00 (123.50–197.00)	198.00 (133.50–407.50)	180.00 (151.00–223.50)	165.00 (146.50–254.00)	**141.00 (139.00–154.00)**	131.00 (129.00–174.00)	70–105
	CX-01	182.00 (178.75–211.25)	221.50 (154.00–351.75)	196.00 (131.00–214.25)	169.50 (143.25–199.25)	**176.00 (166.75–184.25)†**	168.00 (164.00–193.00)	
Anion GAP (mmol/L)	Vehicle	15.50 (12.00–18.00)	23.50 (16.00–26.00)	17.00 (15.00–18.00)	14.00 (11.75–17.00)	17.50 (11.50–19.75)	13.00 (13.00–19.00)	10–20
	CX-01	11.00 (10.25–15.50)	20.50 (15.75–22.00)	14.00 (11.00–15.75)	15.00 (9.50–17.00)	16.50 (14.75–17.25)	16.00 (13.00–17.00)	

Abbreviations: BL, baseline; MAP, mean arterial pressure; HR, heart rate; Na+, sodium; K+, potassium. Data are presented as median (interquartile ranges, IQR). A Mann-Whitney U test was used for comparisons between the vehicle control and CX-01, treatment groups (†, p < 0.05; ††, p < 0.01). An unpaired Mann-Whitney rank test was used to compare individual time points to baseline within the injury group (*, p < 0.05; **, p < 0.01). Statistically significant values are indicated by boldface type.

## 3 Results

### 3.1 CX-01 treatment improves survival in the rat polytrauma model

In this model, we found that CX-01 treatment significantly increased survival rates compared to the vehicle control group (87.5% vs. 33.3%, *p* < 0.05, Figure 1B). In the CX-01 treated group, seven of the eight rats survived to the end of the study, with only one death occurring within the first day. Conversely, only three of the nine untreated control animals survived; five animals died within 24 h post-injury, and one rat died within 48 h post-injury (Figure 1B). Furthermore, untreated control animals experienced a significant drop in body weight starting at 24 h post-injury, which persisted through the end of the study at 72 h. In contrast, CX-01 treatment alleviated this body weight loss, showing a statistically significant difference compared to the untreated control group at the 48 h post-injury (93.7% ± 1.7% vs. 87.5% ± 0.5, *p* < 0.05, Figure 1C).

### 3.2 CX-01 treatment mitigates AKI in the polytrauma rats

**FIGURE 2:**
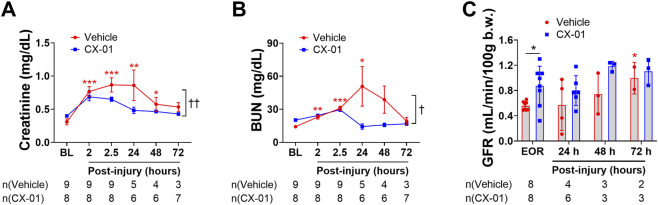
CX-01 treatment improves renal function following trauma-induced AKI. The levels of AKI indicators of creatinine **(A)** and BUN **(B)** levels were measured at baseline (BL), and 2, 2.5, 24, 48, and 72 h post-injury using iSTAT device and presented as mean ± SEM. CX-01 treatment led to a significant reduction in both creatinine and BUN levels compared to the vehicle control group. **(C)** The glomerular filtration rate (GFR) was assessed by measuring the excretion kinetics of FITC-sinistrin in the rats using a MediBeacon optical device. GFR was significantly improved at the end of resuscitation in the CX-01 treated group. Statistical analyses were performed by the linear mixed-effect model for repeated measures, and for the least square means of individual group comparisons. *, *p* < 0.05, the value of individual time point of vehicle vs. CX-01; and †, *p* < 0.05; ††, *p* < 0.01 (black), for the least square means between the groups. An unpaired Mann-Whitney rank test was used to compare individual time points to baseline within the injury group (*, *p* < 0.05; **, *p* < 0.01; ***, *p* < 0.001, red).

Following polytrauma, plasma creatinine levels in untreated animals promptly increased from a baseline of 0.31 mg/dL to 0.77 mg/dL at 2 h and peaked at 0.87 mg/dL by 2.5 h post-injury (>2-fold increase) (Figure 2A). Although levels gradually decreased thereafter, they remained significantly elevated above baseline until 72 h post-injury (Figure 2A). CX-01 treatment significantly mitigated this overall increase in creatinine compared to the untreated control animals (*p* = 0.0082, 95% CI = 0.0070 to 0.2976, Figure 2A). Similarly, CX-01 treatment also led to significantly lower BUN levels (*p* = 0.0212, 95% CI = 2.747 to 18.300, Figure 2B). Critically, a significant improvement in the glomerular filtration rate (GFR), a key indicator of kidney function, was observed at the end of resuscitation (EOR), approximately 2.5 h after the initial CX-01 dose (*p* = 0.0362, Mann-Whitney U = 12, Figure 2C).

### 3.3 CX-01 treatment reduces kidney tissue damage

**FIGURE 3:**
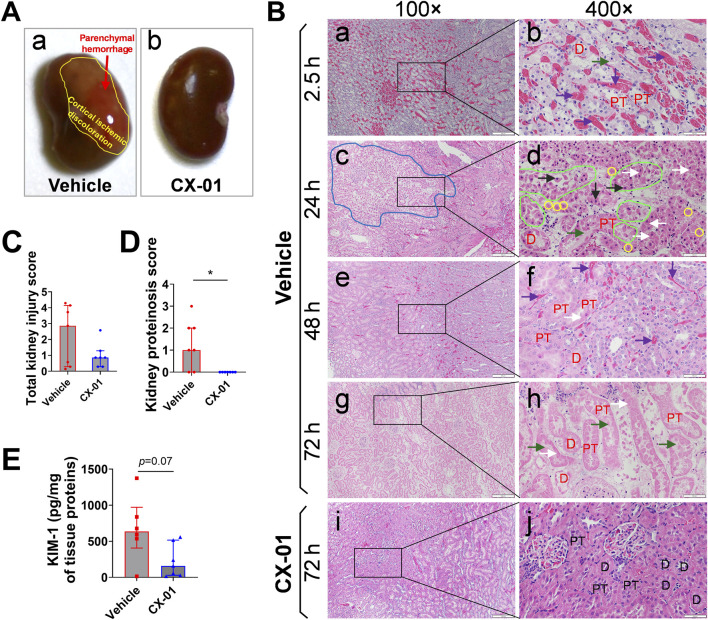
CX-01 treatment reduces tissue damage in the kidney. **(A)** Representative macroscopic kidney images show reduced gross pathological damage in a kidney from a subject treated with CX-01 (b) compared to a vehicle control (a). In the vehicle control, there is a well-demarcated area of pallor (encircled by the yellow line) suggestive of acute infarction, and there are coalescing patches of red within this area (red arrow) consistent with acute hemorrhage. These findings were observed in the vehicle control rats but not in the CX-01 treated rats. **(B)** Representative photomicrographs of histological changes in the kidneys from the vehicle control rats (a–h) and CX-01 treatment rats (i,j). In the vehicle control group, there is increased interstitial hemorrhage and congestion of the peri-tubular vasa recta (purple arrows), both of which were evident in subjects 2.5–48 h post-injury. Tubular epithelial injury involving proximal tubules (PT, red) and distal segments (D, red) is present and persisted through 72 h post-injury. This damage is characterized by tubular epithelial degeneration including loss of epithelial cell polarity and swollen, vacuolated cells, or necrosis characterized by cell shrinkage, sloughing of epithelial cells from the basement membrane into the tubular lumen (green circle), increased cytoplasmic eosinophilia (white arrow), and nuclear pyknosis (black arrow), karyorrhexis, or karyolysis (yellow circle). At 24 h post-injury, a confluent region consistent with established acute tubular necrosis (ATN) was also present (blue circle). Occasionally at 72 h post-injury there were large geographic areas of ischemic necrosis (infarct) characterized by loss of differential staining with retention of architecture (h). In contrast, the CX-01 treated kidney showed a notable preservation of tubular architecture **(i,j)**, with intact proximal tubules (PT, black) and distal tubules (D, black) without evidence of necrosis and only rare minimal degeneration in some kidneys, indicating a significant improvement in overall tubular morphology. Magnification is 100 × for the left panels and 400 × for the right panels. The scale bar represents 500 µm. **(C, D)** Semi-quantitative evaluation of total kidney injury scores and kidney proteinosis scores. The data are presented as median (IQR). Statistical significance was determined using the Mann-Whitney U test. **(E)** Levels of the renal injury biomarker KIM-1, measured by ELISA in tissue lysates, were lower in the CX-01 treatment group. Statistical significance was determined using the Mann-Whitney U test.

Gross pathology observations revealed ischemic damage and hemorrhage in the vehicle control animals, which were not observed in the CX-01 treated animals (Figure 3A). Furthermore, histopathological analysis using H&E staining showed that polytrauma caused severe interstitial hemorrhage and vascular congestion, extensive both proximal and distal tubular epithelial injury, and interstitial inflammatory cell infiltration (Figure 3B). Specifically, tubular epithelial necrosis—characterized by a loss of epithelial cell integrity, cytoplasmic eosinophilia, and nuclear pyknosis/karyolysis—and disruption of the tubular architecture were observed in the vehicle control animals (Figure 3B). In contrast, these histological changes were dramatically reduced in the CX-01 treated animals, which presented with intact proximal and distal tubules and normal tubular morphology (Figure 3B). Semi-quantitative histological scoring revealed that CX-01 largely reduced trauma-induced kidney tissue damage [total kidney injury score 2.86 (0.29–4.14) vs. 0.86 (0.29–1.29)] (Figure 3C). Specifically, CX-01 treatment was associated with a significant reduction of tubular proteinosis [kidney proteinosis score 1.00 (0.00–2.00) vs. 0 (0–0), *p* < 0.05, Figure 3D]. To further validate the extent of kidney tissue damage, the injury biomarker of kidney injury molecule-1 (KIM-1) was quantified in tissue homogenates via ELISA, calculated as a ratio of KIM-1 concentration to total protein concentration in tissue homogenate supernatant. CX-01 treatment resulted in a nearly 75% reduction in median renal KIM-1 expression, which is from 635.7 (406.6–971.8) pg/mg to 159.5 (29.3–516.1) pg/mg (p = 0.07, Figure 3E).

### 3.4 CX-01 treatment reduced trauma-induced lactate elevation and modulates metabolic parameters

**FIGURE 4:**
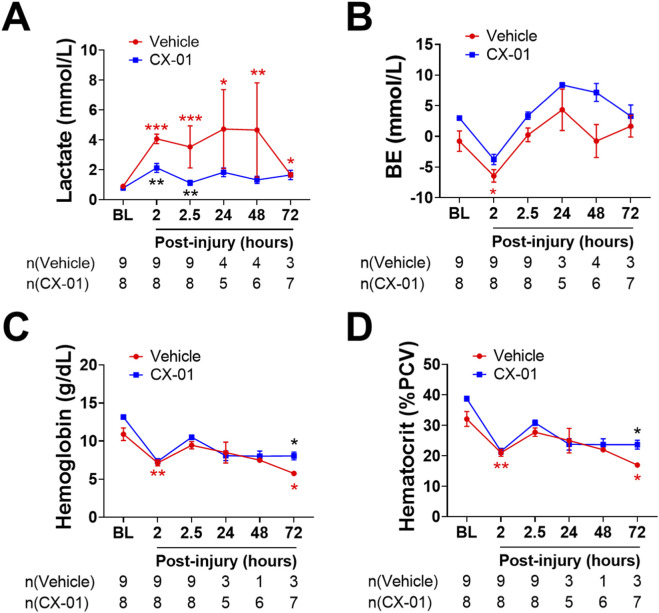
CX-01 treatment alters blood gases and blood chemistry. The blood samples were collected at baseline (BL), and 2, 2.5, 24, 48, and 72 h post-injury for blood gases and blood chemistry analyses. CX-01 treatment significantly reduced lactate level, especially at 2 h post injury **(A)**, and mitigated the elevation of base excess (BE) after polytrauma **(B)**. The hemoglobin **(C)** and hematocrit **(D)** levels both decreased similarly from BL to 2 h post-injury, indicating a comparable amount of blood loss between groups. However, CX-01 treatment significantly increased both hemoglobin and hematocrit levels at 72 h post-injury compared to that of the injury control group. Statistical analyses were performed by the linear mixed-effect model for repeated measures. *, *p* < 0.05 (black), the value of individual time point of vehicle vs. CX-01. An unpaired Mann-Whitney rank test was used to compare individual time points to baseline within the injury group (*, *p* < 0.05; **, *p* < 0.01; ***, *p* < 0.001, red).

Following trauma, the lactate levels were significantly increased at all post-injury time points compared to baseline (BL) (Figure 4A), while base excess (BE) was significantly reduced at 2 h post-injury (*p* = 0.0186, Figure 4B). However, CX-01 treatment significantly attenuated these changes, particularly the lactate levels at two and 2.5 h post-injury (*p* < 0.0001 and *p* < 0.0001 respectively, Figure 4A). Unexpectedly, CX-01 treatment also led to a significant increase in hemoglobin and hematocrit levels at 72 h post-injury (*p* = 0.0238 and *p* = 0.0238, respectively, Figures 4C,D). Additionally, CX-01 treatment appeared to reduce pH at the end of resuscitation, increased glucose at 48 h post-injury, and significantly altered HCO3 and TCO2 levels at early time points post-injury (Table 1).

### 3.5 CX-01 treatment reduces HMGB1 and inflammatory response after trauma

**FIGURE 5:**
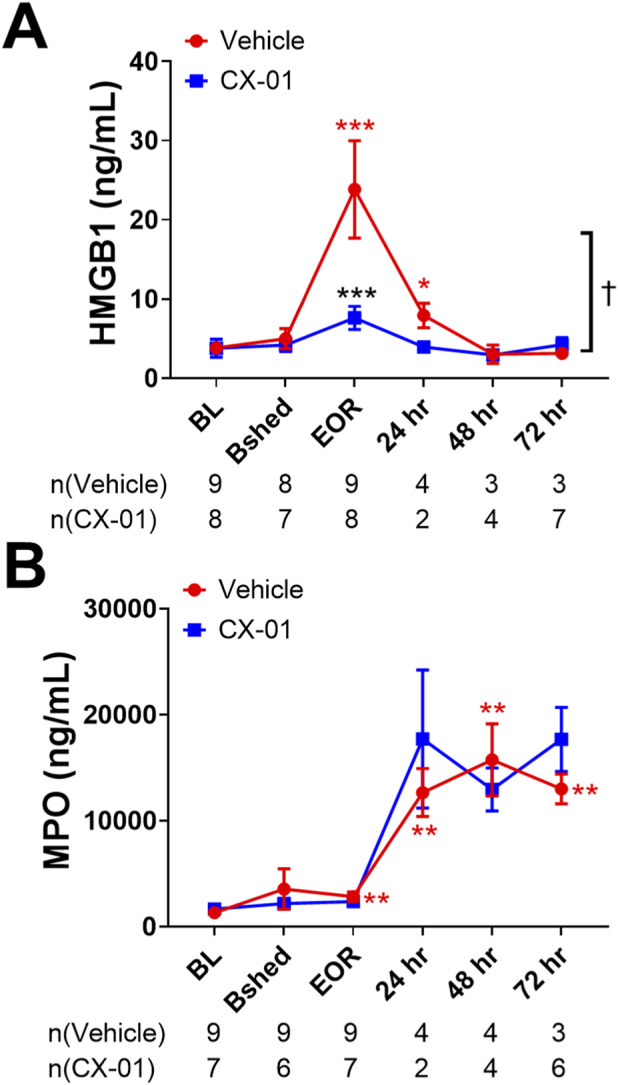
CX-01 treatment selectively reduces circulating HMGB1 but not MPO levels. Plasma samples from the vehicle control and CX-01 treatment groups were analyzed by ELISA to determine circulating levels of HMGB1 **(A)** and MPO **(B)**. Treatment with CX-01 significantly reduced HMGB1 levels, especially at the time point of end of resuscitation (EOR), while MPO levels were not statistically different between the groups. Statistical analyses were performed by the linear mixed-effect model for repeated measures, and for the least square means of individual group comparisons. ***, *p* < 0.001 (black), the value of individual time point of vehicle vs. CX-01; and †, *p* < 0.05 (black), for the least square means between the groups. An unpaired Mann-Whitney rank test was used to compare individual time points to baseline within the injury group (*, *p* < 0.05; **, *p* < 0.01; ***, *p* < 0.001, red).

**FIGURE 6:**
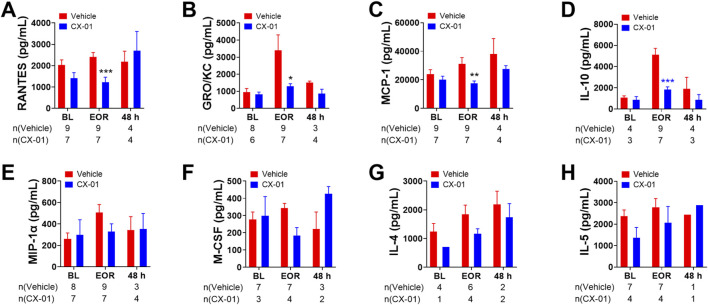
CX-01 treatment reduces circulating inflammatory cytokines levels. Plasma samples were collected from vehicle control and CX-01 treated animals, and a panel of cytokines (RANTES, **(A)**; GRO/KC, **(B)**; MCP-1, **(C)**; IL-10, **(D)**; MIP-1α, **(E)**; M-CSF, **(F)**; IL-4, **(G)**; and IL-5, **(H)**) was analyzed using a Bio-Plex assay. Statistical analysis was performed using the Mann-Whitney test. *, *p* < 0.05; **, *p* < 0.01; ***, *p* < 0.001.

Mechanistically, CX-01 treatment significantly reduced circulating HMGB1 levels especially at EOR (*p* = 0.0097, Mann-Whitney U = 9, Figure 5A), while MPO levels were not statistically different between the treatment and vehicle control groups (Figure 5B). Furthermore, CX-01 treatment broadly attenuated the systemic cytokine response. Specifically, at the end of resuscitation (EOR), levels of RANTES (*p* = 0.0002), GRO/KC (*p* = 0.0229), MCP-1 (*p* = 0.0079), and IL-10 (*p* = 0.0002), were significantly reduced in treated animals compared to the vehicle control group (Figures 6A–D). At the same time point, the levels of MIP-1α, M-CSF, IL-4, and IL-5 were also lower in the treatment group (Figures 6E–H).

### 3.6 Coagulation profile of CX-01 treatment in the rats following polytrauma

**FIGURE 7:**
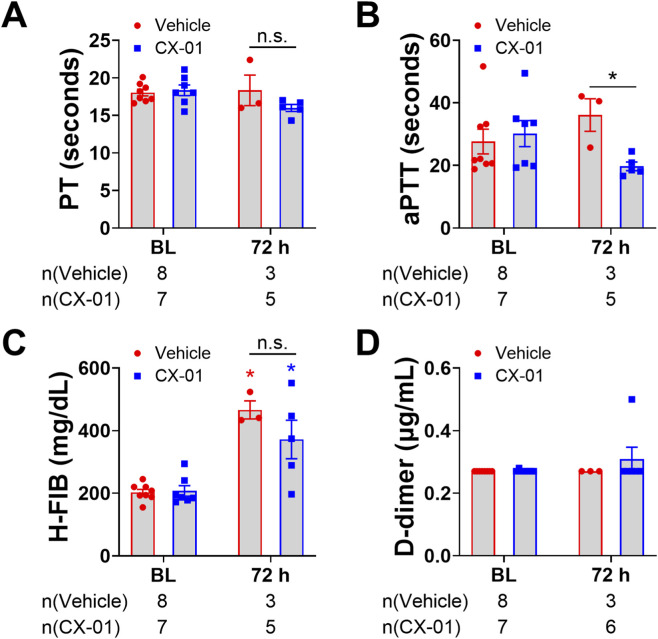
Coagulation profile of CX-01 and vehicle control animals following polytrauma. Citrated plasma was harvested at baseline (BL) and at the end of the study (72 h post-injury) from CX-01 treated and vehicle control groups for coagulation analysis. Prothrombin time (PT; **(A)**), activated partial thromboplastin time (aPTT; **(B)**), high fibrinogen (H-FIB; **(C)**), and D-dimer **(D)** levels were measured using the STAGO STA Compact Max system. An unpaired Mann-Whitney rank-sum test was used to compare 72-h post-injury data to baseline, and to compare the two treatment groups (*, *p* < 0.05; n. s., not significant).

The coagulation profiles of CX-01 treated animals following polytrauma were analyzed using the STAGO STA Compact Max system. Data indicated that both prothrombin time (PT) and activated partial thromboplastin time (aPTT) at the conclusion of the study (72 h post-injury) were comparable to baseline levels in both the CX-01 and vehicle control groups (Figures 7A,B). While PT and aPTT in CX-01 treated animals were slightly lower than those in vehicle controls, these differences did not reach statistical significance. Regarding the high fibrinogen (H-FIB) levels, both the CX-01 and vehicle control groups exhibited significant increases at 72 h post-injury compared to baseline. H-FIB levels were relatively lower in the CX-01 group than in the vehicle control, though the difference had not yet reached statistical significance (Figure 7C). Finally, D-dimer levels for both groups at 72 h remained near baseline values (Figure 7D).

## 4 Discussion

Using a clinically relevant rat polytrauma model, our data demonstrated that CX-01 treatment significantly improved survival, increasing the survival rate from 33.3% in vehicle controls to 87.5%. Additionally, therapeutic treatment with CX-01 significantly mitigated trauma-induced AKI. Specifically, at 2.5 h post-injury, we observed a significantly improved GFR and reduced creatinine levels. The mitigation of AKI was further supported by tissue histological evaluation and tissue AKI biomarker analysis. In the current study, we observed a biphasic pattern of mortality. Based on hemodynamic data, inflammatory response and histological evaluations, we hypothesize that early deaths (<12 h) were predominantly driven by acute cardiovascular collapse and an immediate systemic inflammatory cascade. In contrast, late deaths (>12 h) were likely the result of sustained systemic inflammation progressing to secondary multiple organ failure. Furthermore, our data showed the CX-01 treatment did not alter the coagulation profile when compared to the vehicle controls. Currently there is no anti-inflammatory pharmacological therapy available to treat AKI, besides supportive care (Gonsalez et al., 2019; Pickkers et al., 2022; Ronco et al., 2019). Thus, the data presented here suggest that CX-01 might serve as a potential, safer immunotherapeutic for mitigating trauma-induced AKI, particularly in military prolonged casualty care (PCC) scenarios or civilian critical care settings.

Many studies have revealed that trauma initiates a prompt DAMP-driven inflammatory response (Sharma and Naidu, 2016; Ma et al., 2024; Huang et al., 2024). Due to hemorrhage, ischemia and reperfusion injury (IRI), DAMPs including extracellular cold-inducible RNA-binding protein (eCIRP), extracellular adenosine triphosphate (ATP), heat shock proteins (HSPs), and others are actively or passively released into extracellular space, which can trigger extensive downstream innate immune response (Qiang et al., 2013; Yang et al., 2020; Horner et al., 2023; Ren et al., 2016). Among these DAMPs, HMGB1 has been recognized as one of the key inflammatory mediators, to activate trauma-induced inflammation (Yang et al., 2020; Magna and Pisetsky, 2014; Deng et al., 2019; Wang et al., 1999). We and others using preclinical animal models have demonstrated that HMGB1 significantly increased after blast and hemorrhagic shock (Yang et al., 2022b; Young et al., 2023; Yang et al., 2023b). More directly, Cohen et al., analyzed 169 severe trauma patients with mean of injury severity score (ISS) of 17, and found that HMGB1 was significantly increased at admission to the critical care department (Cohen et al., 2009). The HMGB1 levels are significantly higher in plasma from non-survivors compared to that of survivors (Cohen et al., 2009). Our data, consistent with previous studies, indicate that HMGB1 is promptly activated after severe injury.

HMGB1 activation after trauma injury activates downstream pathways, leading to an extensive inflammatory response, severe cytokine storms, endothelial cell damage, ROS production, coagulopathy, and complement activation, which can ultimately culminate in organ failure and death (Hietbrink et al., 2006; Sauaia et al., 2017; Huber-Lang et al., 2018). HMGB1 activates downstream pathways through its well-characterized receptors. First, disulfide-HMGB1 engages Toll-like receptor 4 (TLR4) to generate pro-inflammatory cytokine release (Yang et al., 2015). Second, extracellular HMGB1 induces inflammatory responses via the receptor for advanced glycation end-products (RAGE), this interaction leads to direct NF-κB activation and subsequent cytokine production (Rauvala and Rouhiainen, 2007). Critically, the activation of HMGB1 correlates with clinical outcomes. Indeed, we have previously shown that HMGB1 correlates with cytokine responses, coagulation dysfunction, and complement activation (Yang et al., 2022b). More directly, in a smoke inhalation and burn injury model, our data showed that HMGB1 activation correlates with the development of ARDS (Young et al., 2023), AKI, and multiple organ failure (MOF) (Yang et al., 2024).

Given its critical role in the pathogenesis of trauma-induced organ failure, targeting HMGB1 might be a promising therapeutic strategy to reduce mortality and morbidity rates in trauma patients. Many laboratories, including ours, have tested whether anti-HMGB1 therapy can improve clinical outcomes, especially in the context of trauma (Yang et al., 2020). Previously, in a rat model of blast and hemorrhage, our group demonstrated that blocking HMGB1 significantly improved survival through the reduced tissue damage in the lung, brain, and liver (Yang et al., 2022b). The data presented here further extends the evidence for the benefits of HMGB1 protect against trauma-induced organ failure. Particularly, this study is the first to demonstrate that CX-01 therapy can mitigate trauma-induced AKI. The relevance of targeting HMGB1 is not limited to trauma; numerous studies have shown it also improves outcomes in infectious contexts like sepsis. For example, Wang and colleagues demonstrated that HMGB1 is a late mediator of endotoxin lethality and that administering neutralizing anti-HMGB1 antibodies could rescue mice from septic shock, even when it was given after the initial insult (Wang et al., 1999).

CX-01, binds to MD2, a component of the TLR4 receptor complex (Rao et al., 2010; Kovacsovics et al., 2018). It thereby blocks the HMGB1-TLR4 interaction, dampening the downstream inflammatory pathways triggered by HMGB1 (Rao et al., 2010; Sharma et al., 2014; Griffin et al., 2014). Previously, our data showed that TLR4 is activated in local tissue during traumatic injury and HMGB1 interacts directly with TLR4 (Young et al., 2023). Theoretically, blocking HMGB1 by CX-01 should not completely block HMGB1 downstream signal pathway activation, as HMGB1 can still signal via other receptors, such as RAGE. Despite this, we observed that CX-01 treatment significantly decreased the inflammatory response, particularly the levels of several cytokines, including RANTES, GRO/KC, and MCP-1, were significantly reduced after treatment. The finding of reducing inflammation after blocking HMGB1 signal is consistent with our previously observations from the blast and hemorrhage rat model (Yang et al., 2022b), and also is consistent with other reports in sterile trauma and infection preclinical models (Wang et al., 1999; Yang et al., 2015; Entezari et al., 2014; Wang L. et al., 2015; Yang et al., 2021). Besides CX-01 inhibition, an alternative approach would be to directly inhibit HMGB1 with a specific monoclonal antibody, which may reduce the side effects of general HMGB1 inhibition. For example, Nakata et al. showed that a murine-derived anti-HMGB1 monoclonal antibody (mAb) decreased plasma HMGB1 levels and reduced inflammatory responses in a murine ischemia-reperfusion model (Nakata et al., 2021). Similarly, neutralizing HMGB1 with this specific mAb also attenuated pulmonary edema and inflammation in a mouse model of acute lung injury (Entezari et al., 2014). Taken together, these data shed light on the potential of direct HMGB1 inhibition for improving outcomes in trauma-induced inflammation and organ failure.

In addition to reducing the inflammatory response, our study also revealed that CX-01 treatment can attenuate the lactate elevation induced by polytrauma. Systemic acidosis is frequently reported in preclinical trauma models and patients, especially when hemorrhagic shock and ischemia disturb glucose metabolism, a condition further worsened by mitochondrial dysfunction (Freitas and Franzon, 2015). Our data indicated that CX-01 treatment leads to a reduction in lactate elevation, especially at 2.5 h post-injury. This may be related to an indirect effect, such as potentially blocking a secondary wave of local cell damage, as well as endothelial and mitochondrial dysfunction, which are often caused by severe trauma (Yang et al., 2022a; Barry and Pati, 2022; Andrianova et al., 2025). Future studies are needed to directly investigate whether CX-01 can prevent or mitigate trauma-induced endothelial and mitochondrial dysfunction.

CX-01 is a modified heparin derivative (Rao et al., 2010). Although its anticoagulant activity has been reduced by over 90% compared to unmodified heparin (Rao et al., 2010), the potential to disrupt the coagulation cascade remains a theoretical concern, particularly at early time points when circulating concentrations may be high. In the current study, we demonstrated that CX-01 did not prolong coagulation times, as evidenced by prothrombin time (PT) and activated partial thromboplastin time (aPTT) values that were comparable to vehicle controls at the end of the 72-h study period. Interestingly, we observed that fibrinogen levels were significantly increased after trauma compared to baseline in both groups. This is likely due to the robust physiological response to acute trauma, as fibrinogen is a well-documented acute-phase reactant (Hayakawa, 2017; Sawamura et al., 2009). This elevation indicates that the animals experienced a sustained, robust systemic inflammatory response following the initial polytrauma. Furthermore, our data showed that D-dimer levels remained near baseline, indicating an absence of pathological systemic clot turnover or hyperfibrinolysis. Collectively, these coagulation profiles suggest that the therapeutic dose of CX-01 used in this study is safe, well-tolerated, and does not induce coagulopathy in a rat polytrauma model.

Several limitations of this study should be acknowledged. First, due to the limitations on blood sampling volume in small animal models, only a limited number of time points were evaluated in our study. The large interval between 2.5- and 24-h post-injury may obscure any important findings on the dynamic changes of inflammatory responses and other processes, such as coagulation. Additional time points, such as at six or 12 h post-injury, would be valuable for more fully characterizing the pathophysiological evolution during this period. Second, we used a pressure-controlled hemorrhage model. While clinically relevant, this design masks any potential beneficial hemodynamic effects of CX-01 treatment, such as an increase in blood pressure. It will be very interesting to see the therapeutic benefits of CX-01 in a volume-controlled hemorrhage model. Third, this study did not include a standard unfractionated heparin control arm. Such a control is necessary for future studies to establish whether CX-01 is superior to heparin in mitigating organ failure and reducing coagulopathy. Fourth, all the data presented here are based on CX-01 early treatment (right after hemorrhage); to better reflect real-world civilian evacuations and military PCC scenarios, future translational studies must evaluate whether CX-01 maintains its protective efficacy when administration is delayed. Fifth, an important limitation of our longitudinal data interpretation is the presence of survivor bias. In the vehicle control group, high early mortality meant that blood and tissue samples collected at later time points (e.g., 48 and 72 h) represented only the most resilient animals. This informative missingness likely underestimates the true severity of the injury in the control cohort at the end of the study. Furthermore, for control animals that died very early (e.g., 2.5 h post-injury), kidney KIM-1 expression likely did not have sufficient time to elevate. Together, these factors artificially depressed the mean injury severity in the control group, which explains why the 75% reduction in KIM-1 did not reach strict statistical significance. Despite this bias, which mathematically narrows the gap between the treated and untreated groups, CX-01 still demonstrated significant therapeutic benefits, underscoring its robust protective effects. Sixth, although our data showed that CX-01 treatment significantly improved several indicators of renal function, the evaluation of additional metrics—such as urine output, formal AKI staging, and other tubular injury biomarkers—would further validate the efficacy of CX-01 in reducing AKI following polytrauma. Finally, while the rat model is relevant, inherent physiological differences exist between rats and humans. Therefore, validation in a large animal model, such as swine, and ultimately in clinical trials, is warranted.

## 5 Conclusion

Acute Kidney Injury (AKI) is a severe complication of trauma, for which effective pharmacological treatments are notably lacking, particularly in modern trauma care. CX-01, if validated, may serve as a potential immunotherapeutic for mitigating trauma-induced AKI, and save the lives for combat casualties and civilian trauma patients.

## Data Availability

The original contributions presented in the study are included in the article/supplementary material, further inquiries can be directed to the corresponding author.
